# The role of silver diamine fluoride as dental caries preventive and arresting agent: a systematic review and meta-analysis

**DOI:** 10.3389/froh.2024.1492762

**Published:** 2024-11-26

**Authors:** Saeed S. Alqalaleef, Rabab A. Alnakhli, Yousef Ezzat, Hamad I. AlQadi, Abdulrahman D. Aljilani, Zuhair S. Natto

**Affiliations:** ^1^ Department of Dentistry, Royal Commission Medical Center, Yanbu, Saudi Arabia; ^2^ Department of Dental Public Health, Faculty of Dentistry, King Abdulaziz University, Jeddah, Saudi Arabia

**Keywords:** carious lesions, dental decay, prevention and control, diamine silver fluoride, review -systematic, primary dentition, permanent dentition

## Abstract

**Background:**

Dental caries is a significant global health concern, impacting around 2.5 billion people worldwide. Conventional methods for managing dental caries have evolved to prioritize preventive measures and minimally invasive treatment alternatives. One of these approaches involves the application of silver diamine fluoride. Although effective, the limited use of SDF is attributed to concerns about potential long-term complications and other adverse effects.

**Objective:**

This systematic review aims to assess the role of SDF in preventing and managing dental caries, evaluating its efficacy and adverse reactions.

**Material and methods:**

This review adheres to PRISMA guidelines. An electronic search was performed on PubMed, Google Scholar, and CENTRAL to include peer-reviewed randomized controlled trials published between 2014 and 2024. The Cochrane risk of bias and GRADE tools were employed to evaluate the trials and ensure the certainty of the evidence. Statistical analysis was conducted to examine the effectiveness of SDF at the individual, tooth, and surface levels.

**Results:**

Findings from 20 randomized controlled trials, which met the inclusion criteria, revealed that participants aged 1–50 showed promising results with follow-up intervals ranging from 1 to 30 months. The caries arrestment rate of silver diamine fluoride (SDF) varied from 25% to 99%.

**Conclusion:**

These results indicate that SDF could be an invaluable tool in combating dental caries, providing a less invasive and potentially more cost-effective alternative to traditional treatment methods. Nonetheless, further research is essential to comprehensively understand the potential of SDF in various settings and to optimize its application in clinical practice.

**Systematic Review Registration:**

https://www.crd.york.ac.uk/, PROSPERO (CRD42024559853)

## Introduction

The global burden of dental caries is a persistent issue transcending age groups and national borders, presenting a considerable challenge to public health systems (1). According to the World Health Organization (WHO), the total number of individuals suffering from dental caries reached approximately 2.5 billion worldwide, affecting about 514 million children (2). In Saudi Arabia, the prevalence of dental caries is estimated to be 75.43% in the primary teeth compared to 67.7% in the permanent teeth (3). This complex condition is marked by the breakdown of dental tissues caused by the acidic bacterial waste found in the oral cavity, which can lead to cavities, discomfort, and, ultimately, the loss of teeth if left untreated (4). Dental caries is a multifactorial disease; besides the influential role of bacteria in the demineralization process of the tooth structure, multiple factors increase caries risk, such as high-sugar diet, poor oral hygiene, xerostomia, and dental appliances, which prevents proper oral homecare (2).

In the past, the primary strategy for addressing dental caries was through reparative treatments, known as “Drill and Fill,” which has been impactful but not to the degree of addressing the entire spectrum of the process of dental caries (5). Nowadays, the focus has shifted more towards preventive measures and less invasive treatment options, including but not limited to maintaining good oral hygiene, pits and fissure sealants, as well as the application of topical fluorides, to reduce caries risk (5). Disrupting bacterial biofilm in the oral cavity to slow down the breakdown of tooth structures can be achieved via proper daily oral hygiene. At the same time, pits and fissure sealants are used to fill deep anatomical structures that act as an environment for bacteria, making oral hygiene measures less effective and eventually rendering the teeth more susceptible to dental decay (6). Topical fluorides come in multiple forms, commonly as sodium fluoride varnish (NaF) and gel, which is primarily classified as an agent to manage dentinal hypersensitivity but also used as a caries preventive agent (7). Fluoride varnish works best in preventing caries by inhibiting demineralization and promoting remineralization. However, it is not capable of arresting it, which withdraws attention to new approaches that have the potential to prevent and arrest dental caries, such as silver diamine fluoride (8).

Silver diamine fluoride (SDF) stands out in this new approach due to its acclaimed ability to halt the progression of existing carious lesions and prevent the emergence of new ones (8). Silver diamine fluoride is composed of silver (antibacterial agent) and fluoride (remineralizing agent) mixed in a liquid base (9). Its application's simplicity and non-invasive nature, along with affordability make SDF an attractive choice, especially in community health initiatives and for individuals with limited access to comprehensive dental care (9).

Nevertheless, SDF is still not as commonly used as its other counterparts. A possible explanation why SDF is still not yet the mainstream, which may be due to unknown long-term complications from chronic exposure of silver, potential staining of the teeth, and several contraindications such as silver allergy and presence of symptomatic teeth and ulcerated tissue (9). Despite its side effects, silver diamine fluoride could be an excellent choice for preventing and arresting dental caries, especially within the socioeconomically disadvantaged population. This highlights the need for an exhaustive review of current evidence to inform clinical practices and effectively shape health policies. Therefore, this systematic review aims to meticulously assess the role of silver diamine fluoride in the prevention and management of dental caries when compared to alternatives, in addition to evaluating its adverse reactions.

## Materials and methods

### Study registry

This systematic review was registered with PROSPERO (CRD42024559853) and was approved by the Medical Ethics and Research Committee of the Royal Commission Medical Center at Yanbu, Saudi Arabia (No: RCYMC-EA-2023-01). This review also adheres to PRISMA guidelines (Preferred Reporting Items for Systematic Reviews and Meta-Analyses).

### Research question

PubMed, Google Scholar, and CENTRAL databases were searched to answer the question: is silver diamine fluoride more effective in arresting and preventing dental caries in primary and/or permanent teeth than the currently practiced methods? PICO question was as follows: P: children and adults; I: silver diamine fluoride. C: placebo, control, and/or other interventions. O: effectiveness in arresting and preventing dental caries.

### Inclusion & exclusion criteria

Inclusion criteria were peer-reviewed randomized controlled trials available in full text, examining children/adults without systemic diseases affecting dental health and reporting results on quantitative methods. Exclusion criteria were studies that combined silver diamine fluoride (SDF) with other preventive methods. Filters applied to the eligibility criteria included articles published in English within the time frame of 2014–2024. The 10-year criteria were selected to balance comprehensiveness, relevance, and feasibility, ensuring that relevant and valuable studies are not excluded while still capturing the most current developments and practices in the field. Grey literature was not included in the research.

### Mesh terms

The following MeSH terms and Boolean operators were used across the searched databases: “Silver Diamine Fluoride” AND “Dental Decay”; “Dental Caries” AND “Preventive Methods”; “Silver Diamine Fluoride” OR “Fluoride Varnish” AND “Dental Decay”; “Silver Diamine Fluoride” AND “Primary Dentition”; Silver Diamine Fluoride; “Silver Diamine Fluoride” AND “Elderly”; “Silver Diamine Fluoride” AND “Dental Caries”; SDF.

### Database search strategy

Two investigators (SA & RA) worked in screening of studies, while the third investigator (YE) disputed any disagreement that might have occurred. The same methodology was utilized during data extraction. The research equation/string for each database is as follows:

#### Pubmed

((Silver Diamine Fluoride AND Dental Decay[MeSH Terms]) OR (Dental Caries AND Preventive Methods[MeSH Terms])) OR (Silver Diamine Fluoride OR Fluoride Varnish AND Dental Decay[MeSH Terms])) OR (Silver Diamine Fluoride AND Primary Dentition[MeSH Terms])) OR (Silver Diamine Fluoride[MeSH Terms])) OR (Silver Diamine Fluoride AND Elderly[MeSH Terms])) OR (Silver Diamine Fluoride AND Dental Caries[MeSH Terms])) OR (SDF[MeSH Terms]).

#### Google scholar

(“Silver Diamine Fluoride” AND “Dental Decay”) OR (“Dental Caries” AND “Preventive Methods”) OR (“Silver Diamine Fluoride” OR “Fluoride Varnish” AND “Dental Decay”) OR (“Silver Diamine Fluoride” AND “Primary Dentition”) OR (“Silver Diamine Fluoride”) OR (“Silver Diamine Fluoride” AND Elderly) OR (“Silver Diamine Fluoride” AND “Dental Caries”) OR (SDF)).

#### CENTRAL

(Silver Diamine Fluoride AND Dental Decay in Title Abstract Keyword OR Dental Caries AND Preventive Methods in Title Abstract Keyword OR Silver Diamine Fluoride OR Fluoride Varnish AND Dental Decay in Title Abstract Keyword OR Silver Diamine Fluoride AND Primary Dentition in Title Abstract Keyword OR Silver Diamine Fluoride in Title Abstract Keyword OR Silver Diamine Fluoride AND Elderly in Title Abstract Keyword OR Silver Diamine Fluoride AND Dental Caries in Title Abstract Keyword OR SDF in Title Abstract Keyword).

The research equations and filters were applied and modified as needed based on the search features of the databases as shown in Table 1.

**Table 1:** Search strategy.

Database	Search equation/string and filters
PubMed	**Equation**: ((Silver Diamine Fluoride AND Dental Decay[MeSH Terms]) OR (Dental Caries AND Preventive Methods[MeSH Terms])) OR (Silver Diamine Fluoride OR Fluoride Varnish AND Dental Decay[MeSH Terms])) OR (Silver Diamine Fluoride AND Primary Dentition[MeSH Terms])) OR (Silver Diamine Fluoride[MeSH Terms])) OR (Silver Diamine Fluoride AND Elderly[MeSH Terms])) OR (Silver Diamine Fluoride AND Dental Caries[MeSH Terms])) OR (SDF[MeSH Terms]) **Filters**: Language: English; Article type: randomized controlled trials; Publication date: 2014–2024; Text availability: full text.
Google Scholar	**Equation**: (“Silver Diamine Fluoride” AND “Dental Decay”) OR (“Dental Caries” AND “Preventive Methods”) OR (“Silver Diamine Fluoride” OR “Fluoride Varnish” AND “Dental Decay”) OR (“Silver Diamine Fluoride” AND “Primary Dentition”) OR (“Silver Diamine Fluoride”) OR (“Silver Diamine Fluoride” AND Elderly) OR (“Silver Diamine Fluoride” AND “Dental Caries”) OR (SDF)) **Filters**: Articles dated: 2014–2024; where the words occur: in the title of the article.
CENTRAL	**Equation**: Silver Diamine Fluoride AND Dental Decay in Title Abstract Keyword OR Dental Caries AND Preventive Methods in Title Abstract Keyword OR Silver Diamine Fluoride OR Fluoride Varnish AND Dental Decay in Title Abstract Keyword OR Silver Diamine Fluoride AND Primary Dentition in Title Abstract Keyword OR Silver Diamine Fluoride in Title Abstract Keyword OR Silver Diamine Fluoride AND Elderly in Title Abstract Keyword OR Silver Diamine Fluoride AND Dental Caries in Title Abstract Keyword OR SDF in Title Abstract Keyword **Filters**: Year: 2014–2024; Language: English

### Assessment of studies

The revised Cochrane Risk of Bias tool for randomized trials (RoB.2) was used to assess the studies' risk of bias (10). Also, the GRADE approach (Grading of Recommendations Assessment, Development, and Evaluation) was used to assess the certainty of evidence (11). GRADEpro GDT software was utilized to summarize and visualize the certainty in evidence (12), while the Robvis visualization tool was used to create a risk of bias plots (13).

### Statistical analysis

To analyze the efficacy of silver diamine fluoride (SDF) in preventing and arresting dental caries, a series of statistical tests were conducted at the individual, tooth, and surface levels using data from the selected randomized controlled trials (RCTs). The statistical analysis was performed using RevMan 5.3 software to ensure robustness and reliability. Descriptive statistics were calculated for all variables, including the number and percentage. These statistics were reported for both the SDF treatment group and the control groups (e.g., placebo, alternative treatments). A meta-analysis was conducted to combine the results from multiple RCTs, providing an overall estimate of the effectiveness of SDF in arresting and preventing dental caries. The meta-analysis included effect size calculations, forest plot, and heterogeneity test. The I^2^ statistic was used to measure the degree of heterogeneity among the included studies. An I^2^ value above 50% indicated moderate to substantial heterogeneity, prompting further investigation into potential sources of variability. Subgroup analyses were conducted based on factors such as concentration of SDF (e.g., 30% vs. 38%). This analysis aimed to identify specific populations or conditions where SDF is most effective. The presence of publication bias was assessed using funnel plots and Egger's test. Any asymmetry in the funnel plot indicated potential publication bias, which was further explored using statistical methods.

## Results

**Figure 1:**
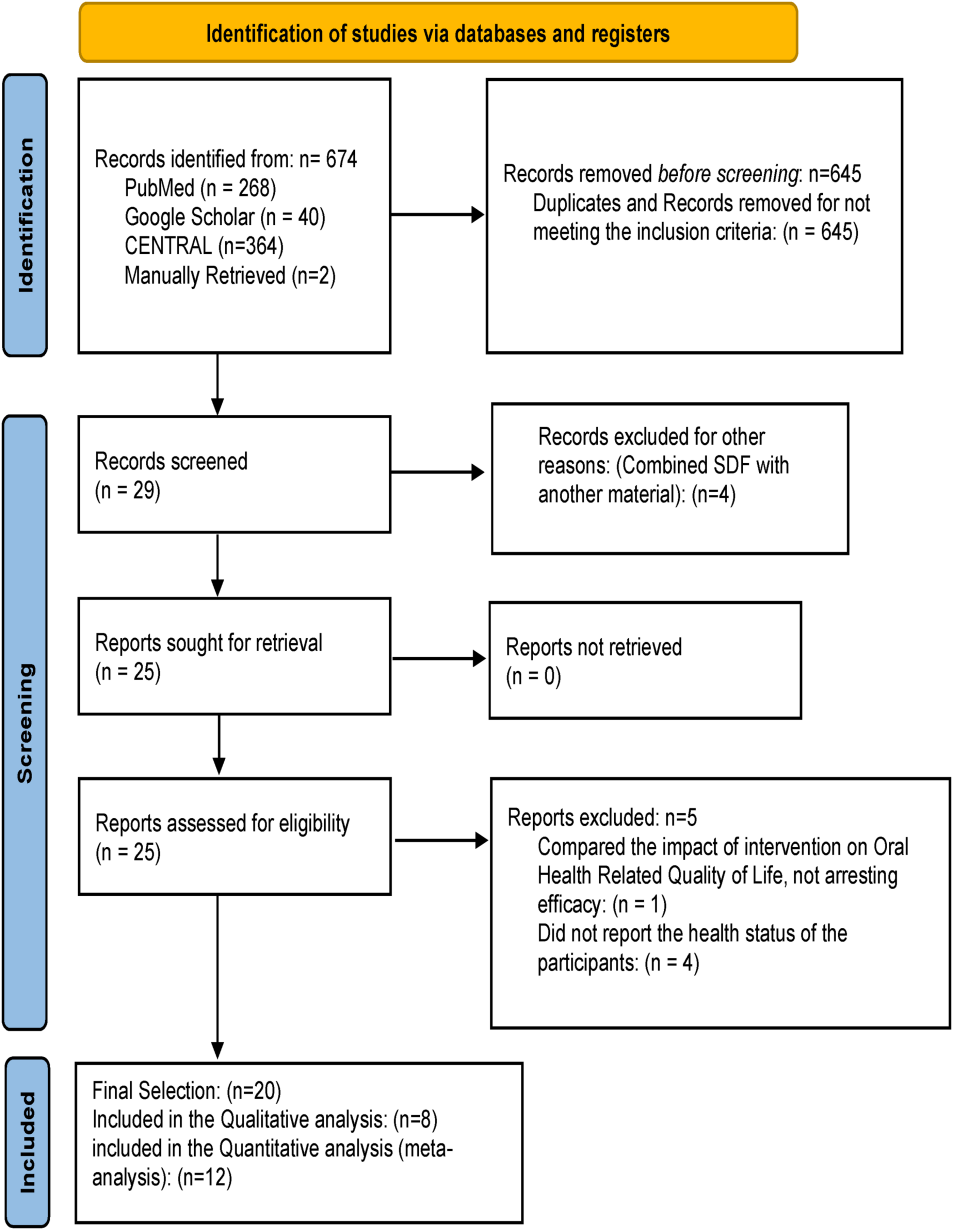
PRISMA study selection flow.

The search resulted in an initial record of 674 articles. A total of 645 duplicate records and studies that did not meet the inclusion criteria were removed before screening. After screening, 4 articles were excluded for combining SDF with another material, resulting in 25 studies for retrieval. All 25 articles were retrieved and assessed against eligibility criteria, and 5 articles were excluded for multiple reasons, as shown in the PRISMA diagram (Figure 1). The final selection included 20 studies, of which 12 were selected for meta-analysis.

Of the selected studies, 18 examined the effectiveness of SDF in arresting dental caries, while the other 2 addressed the preventive efficacy (14, 15). Three studies used 30% SDF (16–18), while the others used 38% SDF. One study only examined permanent teeth (19). The age of participants ranged from 1 to 50 years, with follow-up intervals ranging from 1 day to 30 months. Multiple outcome assessment methods were used, and different criteria were employed to grade lesion severity. Table 2 summarizes the characteristics of the studies.

**Table 2:** Characteristics of studies.

Author/region, date	Sample size	Age, type of dentition	Lesion severity	Follow up	Outcome assessment methods	Blinding	Adverse effect & dropout (%)
Abbas et al./Iraq, 2019 (20)	40 Children	5–7, Primary Dentition	Unknown	2 to 3 Weeks- 6 Months	Unknown	Unknown	Unknown & (5%)
Abdellatif et al./Saudi Arabia, 2021 (21)	90 Children	3–8, Primary Dentition	ICDAS 4,5& 6	6–12 Months	Visual-Tactile Inspection & Radiographs	No	Black Staining & (41.1%)
Abdellatif et al./Egypt, 2023 (22)	220 Children (1606 Lesions)	≤ 4, Primary Dentition	Moderate (ICDAS 3/4) & Advanced (ICDAS 5/6)	6 Months	Visual-Tactile Inspection	Participants, outcome examiner, and biostatistician	Black Staining and 7 events of gum blenching & (9.5%)
Al-Nerabieah et al./Syria, 2020 (23)	119 Children (244 surfaces)	3–5, Primary Dentition	Nyvad Criteria (Unknown level)	3 Weeks-6 Months	Visual-Tactile Inspection	Participants, investigators, and data analyst	Black Staining & (0%)
Al-Nerabieah et al./Syria, 2020 (24)	63 Children (164 teeth)	3–5, Primary Dentition	Advanced (ICDAS 5)	3 Weeks- 3 and 6 Months	Visual-Tactile Inspection	Participants, parents and biostatistician	Black Staining & (0%)
Aly et al./Egypt, 2023 (19)	36 Adults	18–50, Permanent Dentition	Class I Deep Caries (Unknown Criteria	3–6 Months	Visual-Tactile Inspection & Radiographs	Participants, biostatistician, outcome assessor	Black Staining & (0%)
Azuoru et al./Nigeria, 2021 (1)	240 Children	3–10, Primary Dentition	ICDAS 5& 6	2 Weeks- 1 and 3 Months	Visual-Tactile Inspection	Unknown	Black Staining & (2.5%)
Cleary et al./USA, 2022 (25)	98 Children (98 teeth)	2–10, Primary Dentition	ICDAS 5& 6	3–6-12 Months	Visual-Tactile Inspection & Radiographs	Unknown	Black Staining & (30%)
Duangthip et al./China, 2016 (16)	304 Children (1670 Lesions)	3–4, Primary Dentition	Advanced Lesions (ICDAS 5/6)	6–12–18 Months	Visual-Tactile Inspection	Outcome examiner	Black Staining & (9%)
Duangthip et al./China, 2018 (17)	371 Children (2526 Lesions)	3–4, Primary Dentition	Moderate (ICDAS 3/4) & Advanced (ICDAS 5/6)	6–12–18–24–30 Months	Visual-Tactile Inspection	Participants, parents, teachers, providers, outcome examiner	Black Staining & (17%)
Gao et al./China, 2020 (26)	1,070 Children	3–4, Primary Dentition	Unknown	6–12–18–24–30 Months	Visual-Tactile Inspection	Participants, parents, and examiner	Black Staining & (17.7%)
Mabangkhru et al./Thailand, 2020 (27)	302 Children (2,249 Lesions)	1–3, Primary Dentition	Cavitated (Unknown Criteria)	6–12 Months	Visual-Tactile Inspection	Outcome examiner	Black Staining & (12.9%)
Milgrom et al./USA, 2018 (28)	66 Children	2–6, Primary Dentition	Cavitated (Nyvad criteria level 3–6)	14 to 21 Days	Visual-Tactile Inspection	Participants, operator, and examiners	8 events of diarrhea and stomachache & (3%)
Phonghanyudh et al./Thailand, 2022 (29)	290 Children (2,249 Lesions)	1–3, Primary Dentition	ICDAS2 (non-Cavitated) & ICDAS3 (Cavitated)	6–12–18 Months	Visual-Tactile Inspection	Outcome examiner	Black Staining & (15.8%)
Prakash et al./India, 2022 (30)	34 Children (68 teeth)	6–9, Primary	Advanced (ICDAS 5)	6–12 Months	Visual-Tactile Inspection & Radiographs	Outcome examiners and data analyst	Black Staining & (Unknown%)
Quritum et al./Egypt, 2024 (31)	360 Children (1,853 lesions)	4 ≤, Primary Dentition	ICDAS 3 or higher	6–12 Months	Visual-Tactile Inspection	No	Black Staining and gum blenching/pain & (4.7%)
Sirivichayakul et al./Thailand, 2023 (15)	190 children (2,685 surfaces)	4–6, Primary Dentition	Non-Cavitated & Cavitated caries (Unknown Criteria)	6–12–18 Months	Radiograph Examination	Participants, parents, and examiner	Unknown & (17.4%)
Tirupathi et al./India, 2019 (32)	50 Children 159 (Lesions/teeth)	6–10, Primary Dentition	Mount and Hume Classification of caries (Codes 1,2,3)	1–3-6–12 Months	Visual-Tactile Inspection	Participants, operator, and examiner	Black Staining that faded away after 6 months & (7.5%)
Vollú et al./Brazil, 2019 (18)	68 Children (118 teeth)	2–5, Primary Dentition	ICDAS 5& 6	3–6-12 Months	Visual-Tactile Inspection	Outcome assessor	Black Staining and 20 events of burning, bad taste, pain, mouth injury, dissatisfaction with appearance, skin/mouth pigmentation & (22%)
Zheng et al./China, 2023 (14)	688 Children	3–4, Primary Dentition	Unknown	1 Day- 12 Months	Unknown	Participants, parents, and examiner	Unknown (76%)

**Figure 2:**
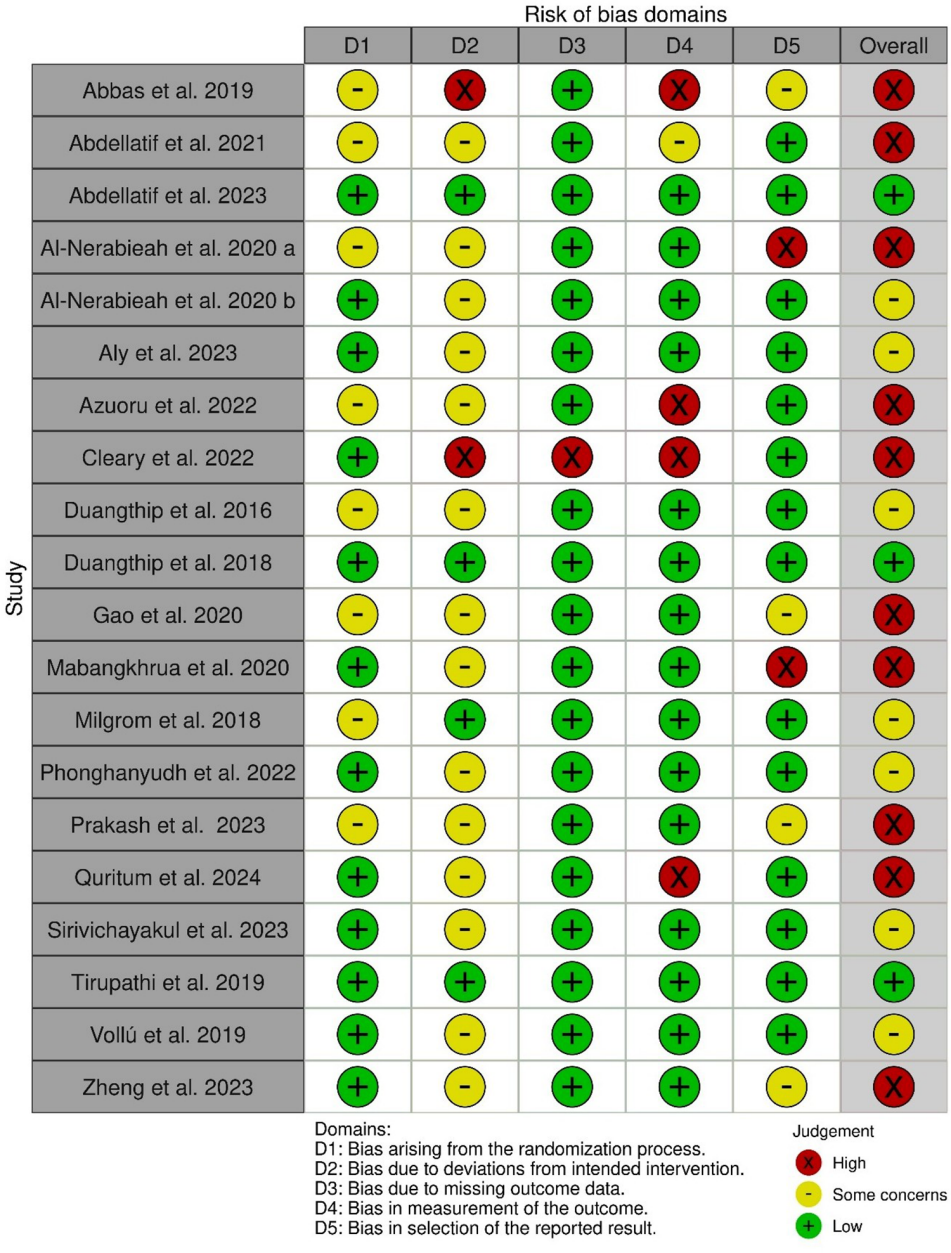
Risk of bias.

**Figure 3:**
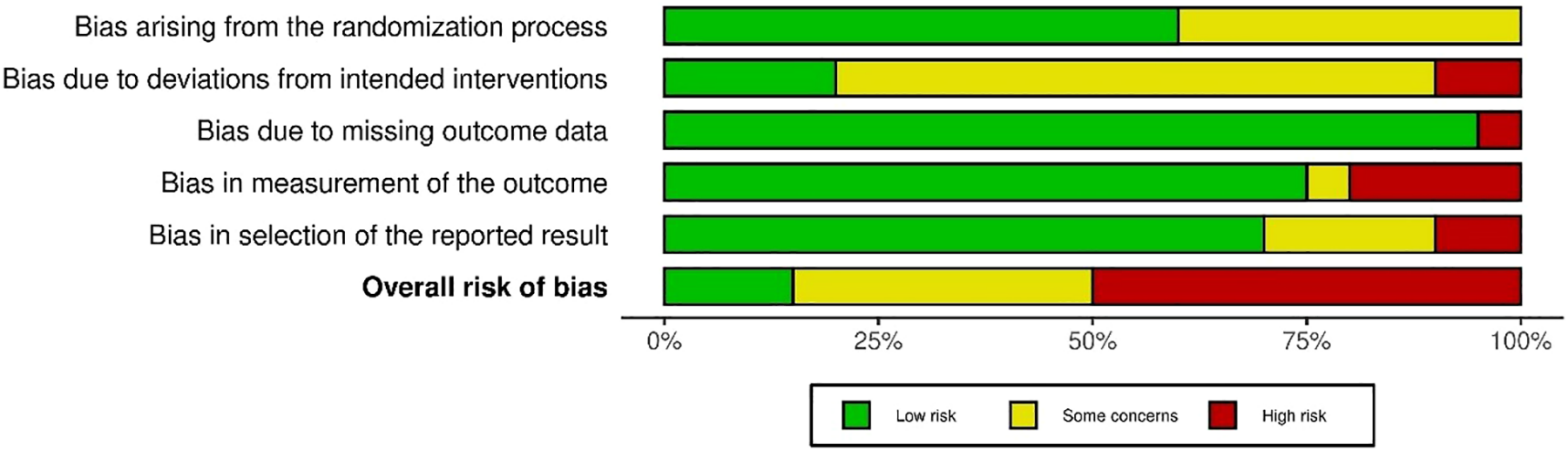
Proportion of risk of bias across domains.

The overall risk of bias assessment (Figure 2) resulted in 3 studies judged as low risk (17, 22, 32), 7 studies with some concerns (15, 16, 18, 19, 23, 28, 29), and 10 studies judged to be at high risk of bias (1, 14, 20, 21, 24–27, 30, 31). Figure 3 shows the proportion of the risk of bias across the domains.

The overall caries arrest efficacy of SDF ranged between 25%- 99% (Table 3) when compared to 27%–58.8% of sodium fluoride varnish (5%NaF) (16, 17, 27, 29, 30), 58.3%- 100% of nano silver fluoride (19, 24, 31), 50.9%–96% of restorative materials (1, 18, 19, 21, 25), and 2.6%–77.7% of placebo and other combined materials (20, 22, 23, 26, 28, 32). The dropout rate ranged from 0% to 76%. When testing for the preventive efficacy of SDF, Zheng et al. showed a reduction of mean decayed surfaces (ds) from 0.7 ± 1.9 to 0.4 ± 1.5 (14). Furthermore, Sirivichayakul et al. showed a caries development rate of 27.2% (15).

**Table 3:** Caries arrestment rate (%).

Author, year	SDF	Comparator/s
Abbas et al., 2019 (20)	38% SDF (1 Application): 31.5%	Placebo (1 Application): 2.6%
Abdellatif et al., 2021 (21)	38% SDF (Bi-Annual): 99%	Glass Ionomer Cement: 94%
Abdellatif et al., 2023 (22)	38% SDF (1 Application): 73.2%	Bi-Annual 38% SDF +5% NaF (1 Application): 77.7%
Al-Nerabieah et al., 2020 (23)	38% SDF (1 Application): 79.5%	Nano Silver Fluoride (1 Application): 67.2%
Al-Nerabieah et al., 2020 (24)	38% SDF (1 Application): 79%	Nano Silver Fluoride + Green Tea Extract (1 Application): 67.4%
Aly et al., 2023 (19)	38% SDF (1 Application): 91.7%	5% Nano Silver Fluoride (1 Application): 100% Control (Composite Restoration): 83.3%
Azuoru et al., 2021 (1)	38% SDF (1 Application): 94.9%	Glass Ionomer Cement: 50.9%
Cleary et al., 2022 (25)	38% SDF (Bi-Annual): 25% (No failure)	Restorative Treatment (Different Materials): 93% (No failure)
Duangthip et al., 2016 (16)	30% SDF (Annual): 40% 30% SDF (3 Weekly Applications): 35%	5% NaF (3 Weekly Applications): 27%
Duangthip et al., 2018 (17)	30% SDF (Annual): - ICDAS 3/4 Lesions: 45% - ICDAS 5/6 Lesions: 48% 30% SDF (3 Weekly Applications): - ICDAS 3/4 Lesions: 44% - ICDAS 5/6 Lesions: 33%	5% NaF (3 Weekly Applications): - ICDAS 3/4 Lesions: 51% - ICDAS 5/6 Lesions: 34%
Gao et al., 2020 (26)	38% SDF (Bi-Annual): 68.9%	25% Silver Nitrate + 5% NaF (Bi-Annual): 70.6%
Mabangkhru et al., 2020 (27)	38% SDF (Bi-Annual): 35.7%	5% NaF (Bi-Annual): 20.9%
Milgrom et al., 2018 (28)	38% SDF (1 Application): 51.7%	Placebo (1 Application): 2.9%
Phonghanyudh et al., 2022 (29)	38% SDF (Bi-Annual): 59.1%	5% NaF (Bi-Annual): 58.8%
Prakash et al., 2022 (30)	38% SDF (Bi-Annual): 77.4%	5% NaF (Bi-Annual): 41.9%
Quritum et al., 2024 (31)	38% SDF (Bi-Annual): - Individual Level: 35.6% - Surface Level: 56.3%	Nano Silver Fluoride (1 Application): - Individual Level: 58.3% - Surface Level: 71.3%
Tirupathi et al., 2019 (32)	38% SDF (1 Application): 71%	5% Novel Nano Silver Fluoride (1 Application): 77%
Vollú et al., 2019 (18)	30% SDF (1 Application): 89%	Glass Ionomer Cement: 96%

**Figure 4:**
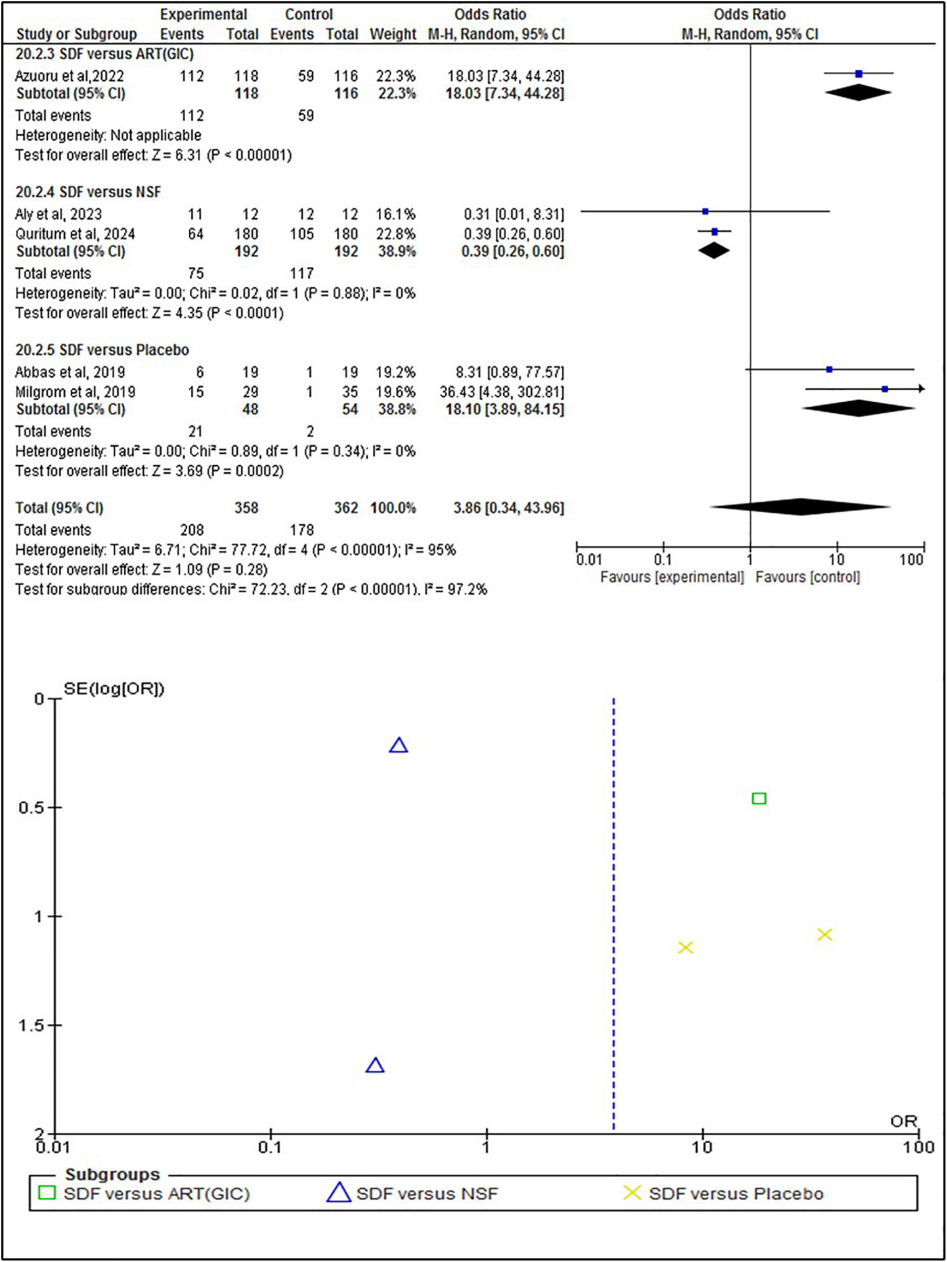
Individual level analysis of caries arrestment.

**Figure 5:**
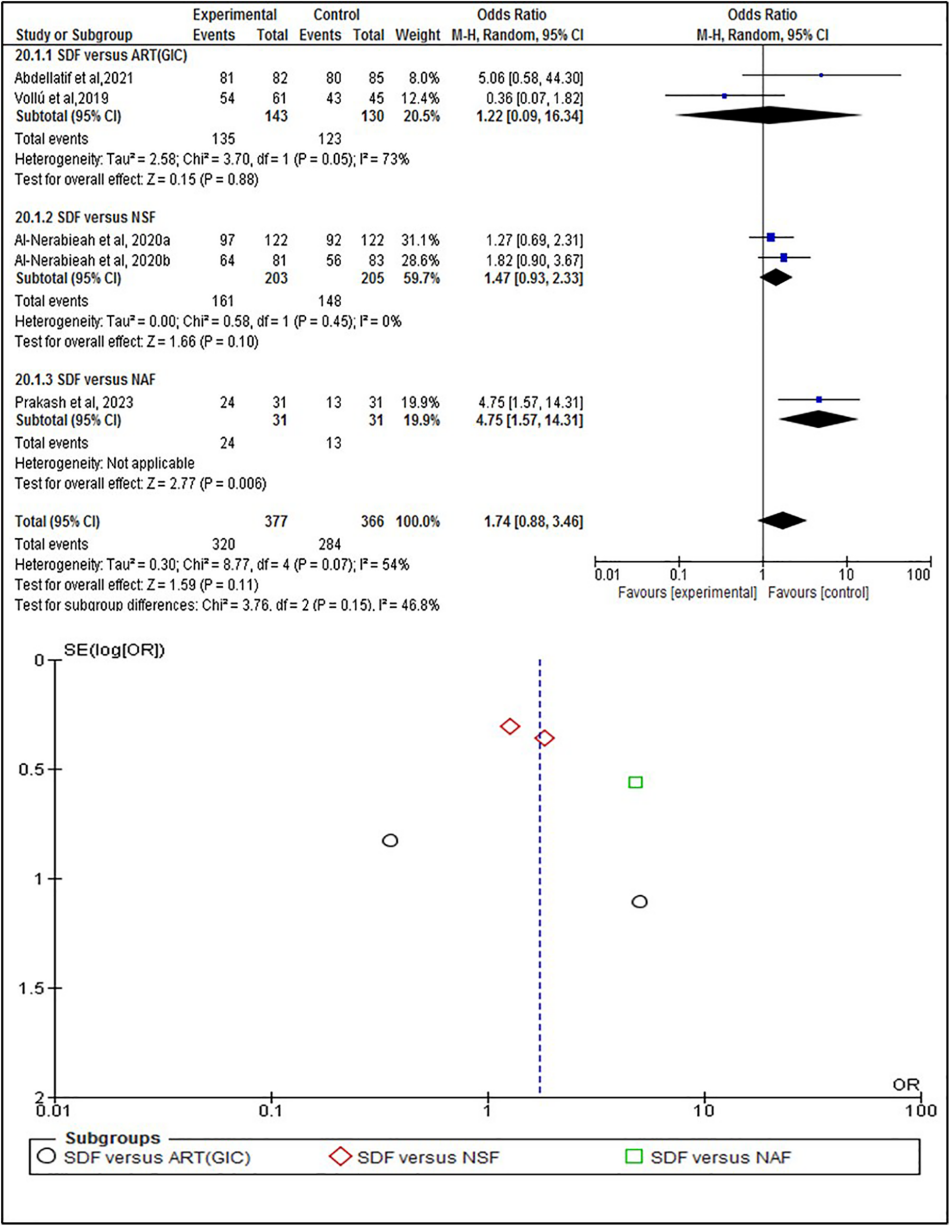
Tooth level analysis of caries arrestment.

**Figure 6:**
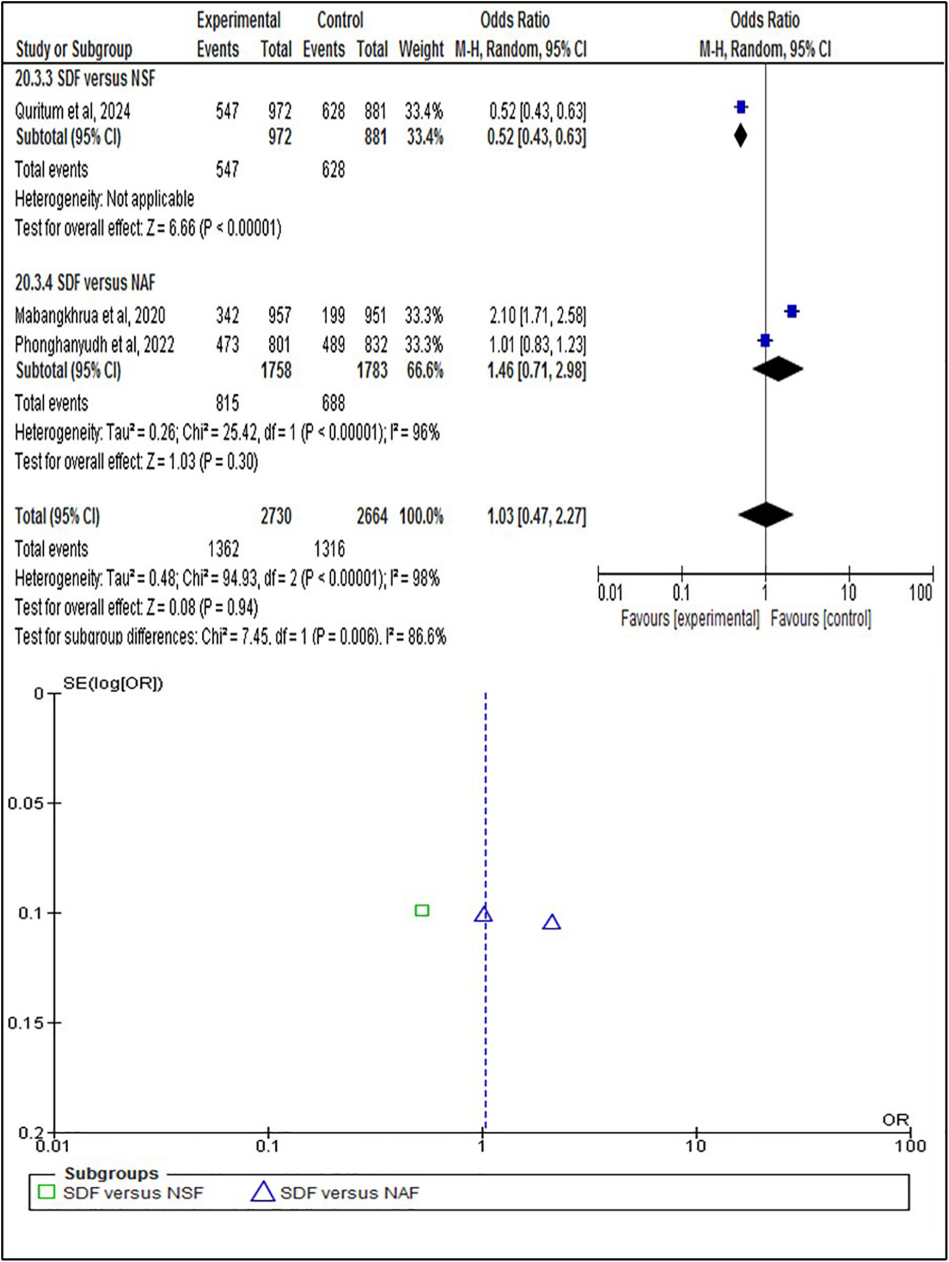
Surface level analysis of caries arrestment.

The statistical analysis was conducted to test the efficacy of SDF on the individual, tooth, and surface levels compared to alternative treatments. At the individual level (Figure 4), SDF treatment demonstrated minor improvements in outcomes compared to other treatments. Specifically, 58% (*n* = 208) of patients who received SDF showed caries arrestment, compared to 49% (*n* = 178) in the control group (*p* = 0.28). No statistically significant difference between these findings that underscores the efficacy of SDF in managing dental caries on a patient-by-patient basis. When evaluated at the tooth level (Figure 5), SDF application was slightly effective in arresting caries progression compared to alternative treatments. In the SDF group, 85% (*n* = 320) of treated teeth remained caries-free, while only 78% (*n* = 284) of teeth in the control group did so (*p* = 0.11); This suggests that SDF is seemingly more beneficial than alternative treatments in maintaining tooth integrity and preventing the spread of caries. The surface level analysis (Figure 6) revealed that SDF-treated surfaces had about the same caries arrestment rate compared to other methods. Specifically, 50% (*n* = 1,362) of SDF-treated surfaces showed caries arrestment, compared to 49% (*n* = 1,316) in the control group (*p* = 0.94). The statistical analysis showed no clear advantage for SDF in preserving the health of individual tooth surfaces, indicating its utility in targeted caries management. No publication bias was detected as per the statistical tests.

## Discussion

**Figure 7:**
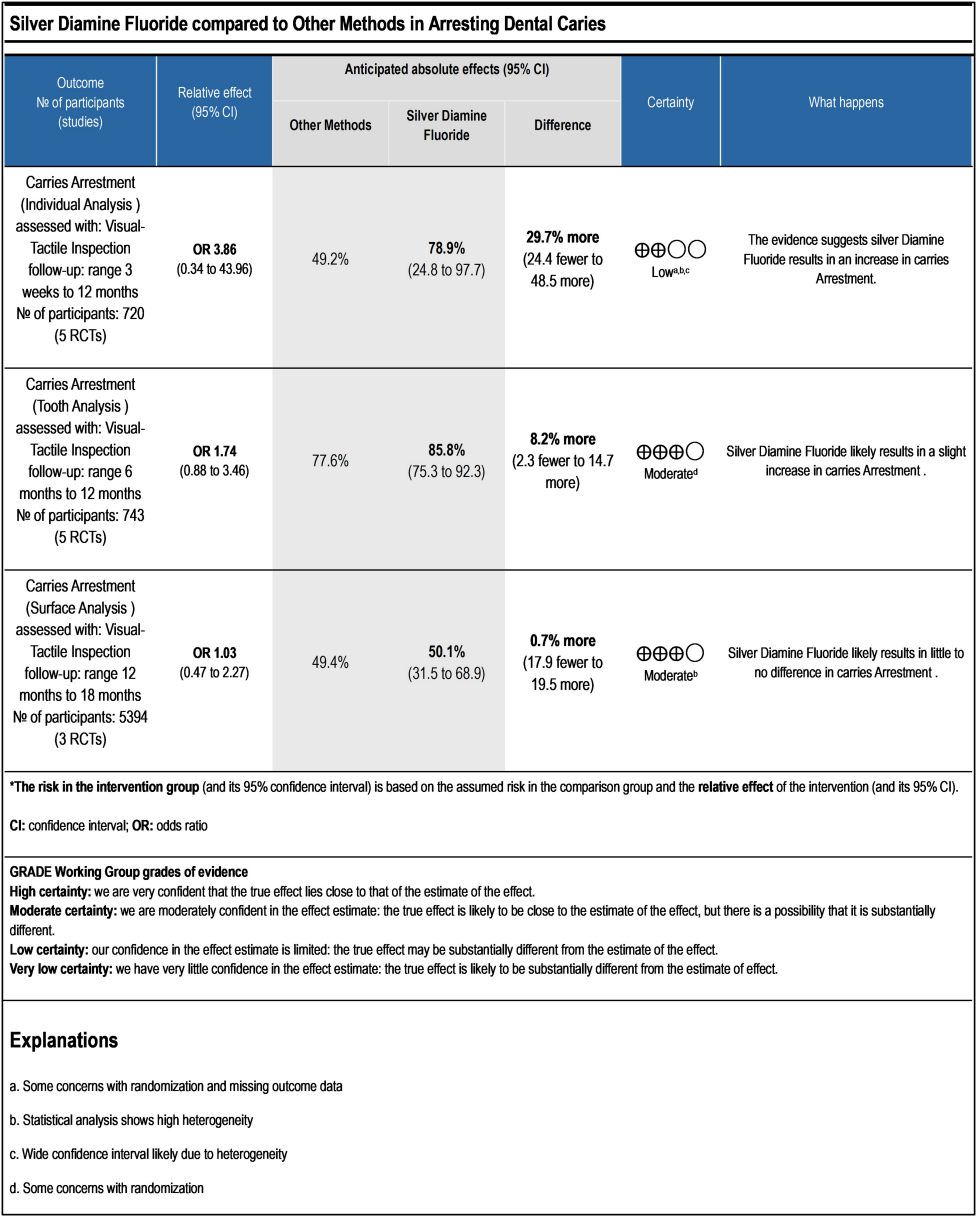
Certainty of evidence (GRADE).

This systematic review primarily focuses on the efficacy of silver diamine fluoride in arresting and preventing dental caries in comparison to alternative approaches. The results of the statistical analysis coincide with the findings of a previous study about the positive effects of SDF (33) and suggest that SDF likely increases caries arrestment when analyzed at the individual level. This was based on a sample of 720 participants from five randomized controlled trials (RCTs), with a follow-up range of 3 weeks to 12 months (Figure 4). When analyzed at the tooth level, SDF likely results in a slight increase in caries arrestment. This was based on a sample of 743 participants from 5 RCTs, with a follow-up range of 6 months to 12 months (Figure 5). However, when analyzed at the surface level, SDF likely results in little to no difference in caries arrestment. This was based on a sample of 5,394 participants from 3 RCTs, with a follow-up range of 12 months to 18 months (Figure 6). These findings suggest that, with a low to moderate level of certainty (Figure 7), the efficacy of SDF in arresting dental caries may vary depending on the level of analysis (individual, tooth, or surface), which highlights the complexity of dental caries arrestment and the need for further research in this field.

Out of the included studies, only one examined the efficacy of SDF in permanent teeth in adults (19), which may give little insight into how SDF could be beneficial in older individuals, especially those with chronic systemic health complications that negatively affect dental health. It is suggested, despite the limited evidence, that SDF is effective in the prevention and arrest of root caries among the elderly (34); however, more studies are needed to explore the full potential of it. With 38% SDF being the most commonly used concentration across the included studies, higher concentrations of SDF are more beneficial. This concurs with previous results that found that an SDF concentration of 38% is superior to 12%, with added benefits if used more frequently, specifically for people with poor oral hygiene and those at high risk of dental caries (35). Additionally, this review provides limited evidence regarding the preventive efficacy of SDF despite the positive results, with only two studies examining it. Yet, Horst and Heima (2019) found SDF to be effective in lowering caries incidence and cost-effective as a preventive agent compared to alternatives such as dental sealants (36).

SDF is not so popular because of the unknown side effects of prolonged exposure to the silver compound. Duangthip et al. (2018) studied the adverse effects of different concentrations of SDF. They concluded that it does not pose significant risks to systemic health except for uncommon mild events of tooth and gum pain, swelling, and bleaching (37). In this review, the majority of studies reported the blackening of the arrested lesions in the SDF groups without major adverse events, except four studies that reported mild events ranging from gum blenching and pain to mild diarrhea and stomachache (Table 1) (18, 22, 28, 31). Therefore, SDF is considered safe to use, especially as instructed. Interestingly, one study reported that stains caused by SDF application started to fade away after six months without knowing the exact cause (32). Furthermore, a systematic review reported the same findings, suggesting that the discoloration of the arrested lesion with SDF could be resolved by applying potassium iodide at the cost of possibly reducing SDF efficacy (33).

Despite the shown efficacy of SDF, there is a disparity in the caries arrest rate across the studies. A possible explanation is the differences in the designs of some studies, acting as limitations that might have affected the accuracy of results. For instance, dental radiographs aid in diagnosing and assessing the progression of carious teeth when clinical examination is deemed not feasible (38). In the current review, only five studies utilized radiographs during assessments (15, 19, 21, 25, 30), thus yielding more accurate results when compared to studies that used only visual-tactile inspection. The majority of studies reported no attempts to remove the carious lesion before applying SDF except Aly et al. (2023), in which deep carious lesions were excavated when needed (19), hence implying that the arrestment might not have been entirely the result of SDF application but rather the excavation process itself. Another factor that might have affected the quality of results is detection bias, which pertains to the study design. Besides the staining of the arrested lesion caused by SDF, blinding in some studies might not have been possible due to the nature of intervention used as restorative materials, making it easier for the operators and outcome assessors to differentiate between groups, increasing the likelihood of detection/observer bias to occur.

The SDF mechanism of action is multifaceted, involving antibacterial properties, remineralization effects, and inhibition of organic matrix degradation (39). The silver ion in SDF is bactericidal, which can kill bacteria or interfere with their metabolic processes, as well as its ability to inhibit the formation of cariogenic biofilms (39). SDF promotes the remineralization of hydroxyapatite in enamel and dentine, the mineral components of teeth. This is achieved through the formation of silver phosphate and calcium fluoride when SDF reacts with hydroxyapatite. The subsequent dissolution of fluoride and calcium facilitates the formation of fluorapatite, a less soluble and more acid-resistant form of hydroxyapatite. This process helps to strengthen the tooth and protect it from further decay (39). Dentine, a component of teeth, contains a significant amount of organic material, primarily type I collagen. SDF has been shown to protect this collagen from degradation, a key factor of tooth decay. This is achieved through the inhibition of matrix metalloproteinases (MMPs) and cysteine cathepsins, enzymes that contribute to the degradation of collagen (39). SDF is a cost-effective option for managing dental caries, especially in young children. It has been shown to reduce dental care expenditures significantly by averting more expensive caries treatment options. For instance, the incremental cost-effectiveness ratio (ICER) of SDF is dominant, meaning it is more cost-effective compared to standard care. The cost-effectiveness of SDF ranges from 74.8% to 100%, making it a highly favorable option for dental caries management (40). This systematic review has some limits in that it pertains to examining only manuscripts published in English and open-access databases, which may have limited the search results, with the possibility of eliminating valuable studies. Additionally, in this review, only studies that examined medically healthy participants were selected per inclusion criteria. Thus, further studies are needed to assess SDF efficacy in people with systemic diseases that may affect dental health.

## Conclusion

This systematic review has provided valuable insights into the efficacy of silver diamine fluoride (SDF) in arresting and preventing dental caries. The findings of this review contribute to the growing body of evidence supporting the use of SDF as a viable alternative to traditional caries management approaches. However, it also highlights the need for further research to fully understand the potential of SDF in different contexts and to optimize its application in clinical practice. Future studies should aim to explore the factors that may influence the efficacy of SDF, such as the severity and location of caries and systemic diseases that affect dental health. In light of the high prevalence of dental caries globally and in Saudi Arabia, the findings of this review have significant implications for public health. SDF could be a valuable tool in the fight against dental caries, offering a less invasive and potentially more cost-effective alternative to traditional treatment methods, especially within rural and underprivileged communities with less dental care access. However, the implementation of SDF in clinical practice should be guided by rigorous evidence-based protocols to ensure its safe and effective use.

## Data Availability

The original contributions presented in the study are included in the article/Supplementary Material, further inquiries can be directed to the corresponding author.
